# Effect of durations and pressures of cupping therapy on muscle stiffness of triceps

**DOI:** 10.3389/fbioe.2022.996589

**Published:** 2022-11-17

**Authors:** Yameng Li, Pu-Chun Mo, Sanjiv Jain, Jeannette Elliott, Adam Bleakney, Shaojun Lyu, Yih-Kuen Jan

**Affiliations:** ^1^ Department of Kinesiology and Community Health, University of Illinois at Urbana-Champaign, Urbana, IL, United States; ^2^ College of Physical Education and Sports, Beijing Normal University, Beijing, China; ^3^ Department of Physical Medicine and Rehabilitation, Carle Foundation Hospital, Urbana, IL, United States; ^4^ Disability Resources and Educational Services, University of Illinois at Urbana-Champaign, Champaign, IL, United States

**Keywords:** cupping therapy, cupping pressure, cupping duration, dose response, elasticity, elastography, and stiffness

## Abstract

Cupping therapy has been used for the alleviation of muscle soreness in athletes. However, clinical studies of cupping therapy show conflicting results. Lack of standardized guidelines of the dose-response relationship of cupping therapy, such as appropriate cupping duration and negative pressure, limits the adoption of cupping therapy in clinical practice. The objectives of this study were to investigate the effect of various pressures and durations of cupping therapy on reducing muscle stiffness. The 2 × 2 factorial design with the repeated measures and counterbalanced design was used to test four cupping protocols, including two negative pressures at −225 and −300 mmHg and two durations at 5 and 10 min, in 12 healthy young people. B-mode and elastographic ultrasound was used to assess muscle stiffness of the triceps before and after cupping therapy. The region of interest of elastographic image was divided into the superficial and deep layers for assessing the effect of cupping therapy on stiffness of various depths of the triceps. Normalized stiffness was calculated as a ratio of pre-cupping stiffness divided by post-cupping stiffness of each participant. The two-way analysis of variance (ANOVA) was used to examine the main effects of the pressure and duration factors and the interaction effect between the pressure and duration factors. The results showed that there were no interactions between the pressure and duration factors (overall layer *p* = 0.149, superficial layer *p* = 0.632, and deep layer *p* = 0.491). The main effects of duration of the overall, superficial and deep layers were *p* = 0.538, *p* = 0.097 and *p* = 0.018, respectively. The results showed that 10-min cupping at -300 mmHg is more effective on reducing stiffness of the deep layer of the triceps compared to 5-min cupping (*p* = 0.031). This study provides the first evidence that the dose of cupping therapy could significantly affect changes of triceps stiffness and the deep layer of the muscle is more sensitive to cupping therapy compared to the superficial and overall layers.

## Introduction

Cupping therapy can be described as an ancient medical technique that uses cups placed over different parts of the body or specific areas (meridians or acupuncture points) of the skin to create negative pressure through suction (Bhikha et al., 2008; Furhad and Bokhari, 2022). Cupping therapy has been widely used for the alleviation of musculoskeletal pain of the neck, shoulder, and back (Trofa et al., 2020; Hou et al., 2021c; Hasbani et al., 2021). The lack of standardized application guidelines, such as appropriate treatment duration, cup size, and negative pressure, and the mechanism limit the adoption of cupping therapy in clinical practice (Stephens et al., 2020; He et al., 2021) (Zhou et al., 2020; Jan et al., 2021).

Various mechanisms have been proposed to explain the potential beneficial effects of cupping therapy (Cao et al., 2012; Al-Bedah et al., 2019; Hou et al., 2021a). The mechanical effect of cupping therapy on increasing local blood flow and stretching underlying soft tissue may explain benefits of cupping therapy (Lowe, 2017) (Al-Bedah et al., 2019; Hou et al., 2021a). These benefits may partly support the reason that cupping therapy is a popular technique used by athletes to reduce muscle soreness and improve performance (Trofa et al., 2020). However, the effect of cupping therapy on the musculoskeletal system (e.g., muscles) has not been fully investigated (Roostayi et al., 2016; Jan et al., 2021).

The muscle is a hierarchical structure and is composed of force generating sarcomeres arranged in the bundles of myofibrils, fibers and fascicles (Lieber et al., 2017). This hierarchical structure determines the muscle’s mechanical behavior including stiffness and elasticity (Bilston and Tan, 2015). Elasticity of the muscle is considered as an important aspect of the muscle function. According to Hill’s classic work in the 1930s, the elastic behavior of the muscle affects the change of length and rate o muscle contraction, and the total force capability of the muscle (Lieber et al., 2017; Herzog, 2019). Muscle stiffness is defined as the proportional relationship between compression and deformation (Creze et al., 2018; Bastijns et al., 2020; Jan et al., 2021). A series of experiments support that muscle stiffness is linearly related to both active and passive muscle forces (Hug et al., 2015). Muscle performance is also affected by metabolic and other systemic disorders. Bensamoun et al. found that hyperthyroid patients had significantly less stiffer thigh muscles than healthy controls (Bensamoun et al., 2007). Abnormal muscle stiffness can be found in patients with neuromuscular impairments or musculoskeletal conditions, such as people with muscle soreness (Costa et al., 2018; Boulard et al., 2019). Estimation of individual muscle stiffness of a localized area provides an opportunity for new insights into changes in muscle mechanical properties on the progression of musculoskeletal and neurological disease and rehabilitation outcome (Hug et al., 2015; Bastijns et al., 2020).

Interventions that aim to alter muscle stiffness are common in physical therapy and traditional Chinese medicine (e.g., massage, exercise, and stretching). The benefits of reducing muscle stiffness are associated with reducing muscle fatigue and soreness after exercise and physical activity (Nakamura et al., 2020; Hou et al., 2021b). In clinical and sports settings, static stretching is usually performed in athletes to decrease passive muscle stiffness (Nakamura et al., 2020; Hou et al., 2021b). Green et al. found that after 48 h of a bout of eccentric exercise induced by walking backwards on a treadmill, stiffness of the calf muscles of volunteers was 21% higher than that before exercise (Green et al., 2012). Research also demonstrated that 7-min massage effectively decreased muscle stiffness (Eriksson Crommert et al., 2015). Cupping therapy is another mechanical intervention like massage and stretching, which applies mechanical forces on the soft tissue (He et al., 2021). Cupping therapy is easier to apply compared to massage and stretching that require extensive training of a clinician. Recent research starts to investigate the biomechanical responses of muscles after applying cupping therapy, and the efficacy of cupping therapy on treating musculoskeletal conditions (Chiu et al., 2020; Jan et al., 2021). For example, cupping therapy with a negative pressure of 400 mmHg for 15 min each time, twice a week, for 4 weeks can be used to improve functional recovery and health in athletes with trapezius myofascial pain syndrome (Chiu et al., 2020). One of the benefits of cupping therapy is considered to be the reduction of muscle stiffness (Jan et al., 2021). Cupping therapy also allows patients with low back pain to regain functional movement in a timely manner by reducing pain and muscle tenderness and improving range of motion (ROM) (Markowski et al., 2014). These mechanical effects associated with cupping therapy may reduce muscle stiffness, and muscle pain, and improve muscle flexibility and range of motion (Jan et al., 2021).

The magnitude of negative pressure used in cupping therapy is an important issue that has been discussed in various studies. The negative pressure can be light (between −100 and −300 mbar; 1 mbar = 0.75 mmHg), medium (between −300 and −500 mbar), and strong (above −500 mbar) (Tham et al., 2006; Moura et al., 2018; Wang et al., 2020). The medium negative pressure is often used for painful conditions of the musculoskeletal system (Moura et al., 2018). The response of the autonomic nervous system to cupping therapy on the back were dependent on with the magnitude of the applied negative pressures (−100, −300, and −500 mmHg). For example, negative pressure at -100 mmHg did not significantly change the heart rate variability (HRV), but negative pressure at both −300 and −500 mmHg caused a significant improvement in HRV (Tang et al., 2019). Previous research demonstrated that −300 mmHg was more effective on increasing skin blood flow compared to −225 mmHg (Wang et al., 2020). However, negative pressure at −450 mmHg may damage soft tissue, and negative pressure below −150 mmHg may be too small to induce a beneficial response of soft tissue (Heinemann et al., 1997). According to a finite element modeling study performed by Tham et al., increasing the negative pressure can increase the tensile stress for pulling deeper tissues into the cup (Tham et al., 2006). Although the general principle of the negative pressure of cupping therapy has been established for clinical practice, it is unclear what ranges of negative pressure could reduce muscle stiffness.

The duration of cupping therapy is another factor that may affect the efficacy of cupping therapy (Wang et al., 2020). The cup is typically left on the skin for 5, 10, or longer minutes. However, most studies used a duration of 10 min (Tham et al., 2006; Roostayi et al., 2016; Chiu et al., 2020). Jan et al. demonstrated that stiffness of the deep layer of the muscle significantly decreased after the application of negative pressure at −300 mmHg (−0.04 MPa), for 5 min in healthy volunteers (Jan et al., 2021). After daily 10-min cupping therapy for 12 days, the skin stiffness and ultimate tensile strength of Wistar Rats reduced (Roostayi et al., 2016). A research study showed that a shorter duration (5 min) caused a larger peak and total skin blood flow compared to a longer duration (10 min) (Wang et al., 2020). However, there is no specific guideline on choosing a duration for reducing muscle stiffness.

The mechanical behavior of the skin and muscle can be characterized by using elastographic ultrasound (Bensamoun et al., 2007; Costa et al., 2018; Boulard et al., 2019). The advantage of ultrasound imaging technique is due to its non-invasive and relatively low cost compared to other imaging techniques (eg. MRI) (Hendriks, 2001). Elastographic ultrasound is a relatively new method to assess soft tissue mechanical property, and has two types of devices including strain elastography and shear wave elastography (Ophir et al., 1991; Garra, 2015). Studies have demonstrated that both strain elastography and shear wave elastography may be used to improve diagnosis of musculoskeletal impairments and document efficacy of rehabilitation interventions (Garra, 2015). Strain elastography is particularly useful on detecting the focal change of mechanical property of soft tissues (Garra, 2015). This feature can be used to compare the change in muscle stiffness after cupping therapy.

Overall, it remains largely unknown about the dose response of cupping therapy, including the values of negative pressure and duration h on reducing muscle stiffness. In order to examine whether different pressures and durations of cupping therapy affect the change in muscle stiffness, the authors proposed to use strain elastography to compare the change in muscle stiffness after various pressures and durations of cupping therapy. To the best of our knowledge, this is the first study to explore the effect of various pressures and durations of cupping therapy on muscle stiffness. We hypothesized that the change in triceps muscle stiffness is dependent on the pressure and duration of cupping therapy and the response from the deep layer of the triceps muscle would be more significant compared to the superficial layer of the triceps muscle.

## Methods

The 2 × 2 factorial design with repeated measures was used in this study. The main effects included the pressure and duration factors and their interaction effect (pressure x duration). The four protocols of cupping therapy, including two negative pressures at −225 and −300 mmHg and two durations at 5 and 10 min, were tested in this study. The specific protocols of cupping therapy were (A) −225 mmHg for 5 min (B) −225 mmHg for 10 min (C) −300 mmHg for 5 min, and (D) −300 mmHg for 10 min. The counterbalanced design was implemented to offset the order effect. Each protocol was separated by 2–4 days. The specific test orders of four protocols of cupping therapy in all participants are shown in Table 1.

**TABLE 1:** The counterbalanced order of four cupping therapy protocols used in this study, including −225 mmHg × 5 min (B) −225 mmHg × 10 min (C) −300 mmHg × 5 min, and (D) −300 mmHg × 10 min.

Subjects	The order of four protocols of cupping therapy
N01	A, B, C, D
N02	B, A, C, D
N03	A, B, D, C
N04	B, A, D, C
N05	C, D, A, B
N06	D, C, A, B
N07	C, D, B, A
N08	D, C, B, A
N09	A, D, B, C
N10	B, C, A, D
N11	A, D, C, B
N12	B, C, D, A

### Cupping protocols

Cupping pressure and duration: There were no standardized guidelines on choosing a specific value of pressure and duration of cupping therapy for reducing muscle stiffness. In this study, the selection of negative pressure of cupping therapy between −225 and −300 mmHg was based on the commonly used setting and our preliminary studies (Heinemann et al., 1997; Wang et al., 2020). The selection of duration of cupping therapy between 5 and 10 min was also based on clinical practice (Wood et al., 2020). The use of four protocols of cupping therapy would cover most used intensities of cupping therapy (Wang et al., 2020).

Cupping device and cup size: Regarding the suction method to create negative pressure, an electronic negative pressure device was used for providing negative pressure inside the cup across all cupping protocols in all participants (Moura et al., 2018). The use of an electronic negative pressure device usually do not involve bloodletting and is a safer procedure. The diameter of cupping cup is regarded as an important factor in cupping therapy. The results of some studies indicate that increasing the cup diameter may cause larger stresses at different tissue layers (Hendriks, 2001; Tham et al., 2006; Jan et al., 2021). According to those findings, the cup diameter in this study was 45-mm (the inner diameter as 45 mm and the outer diameter as 53 mm).

Cupping site: The site for cupping therapy was chosen at the Xiaoluo acupoint (SJ12) of the dominant arm (i.e., dominant arm). SJ12 is located at the triceps and is used to relieve the pain of shoulder and upper limb, and our preliminary research have confirmed that cupping at SJ12 is effective for improving skin blood flow and reducing muscle stiffness (Wang et al., 2020; Jan et al., 2021). The use of 45-mm cup would ensure a large cupping area to cover the location of SJ12.

### Participants

The inclusion criteria included: healthy people aged 18–30 years without any diagnosed diseases. The exclusion criteria included: non-blanchable response of the red skin areas over the triceps (dominant side); open wounds, scar or tattoo over the tested area; any cardiovascular diseases; and smokers. The participants were recruited through flyers and word of mouth methods from the University of Illinois. Each participant signed the informed consent approved by the University of Illinois at Urbana-Champaign Institutional Review Board (#20334).

### Procedures

**FIGURE 1:**
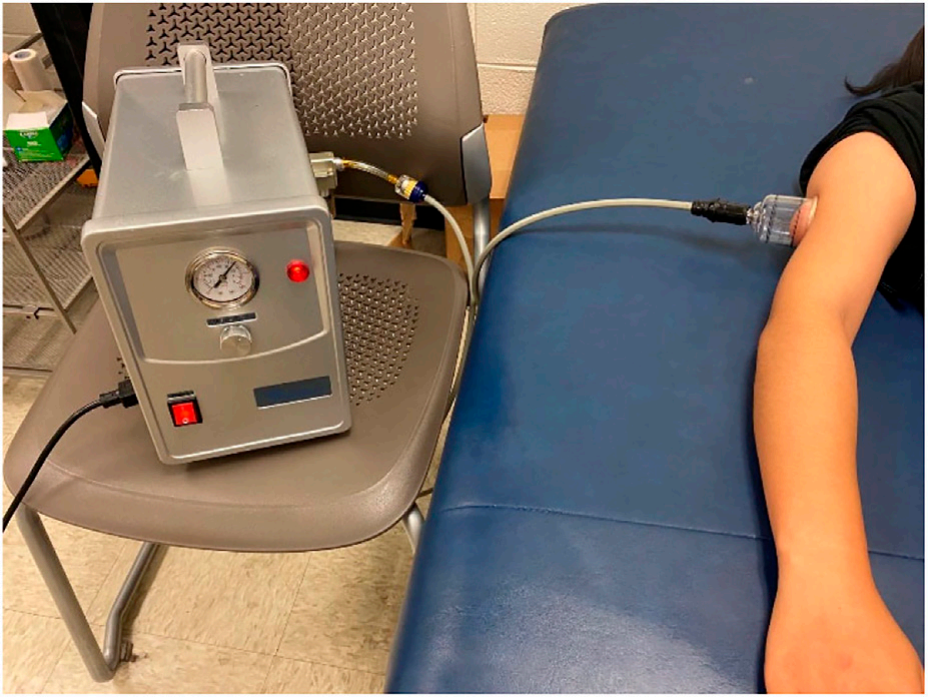
Photograph of the electronic negative pressure device (cupping device) and the setup of the cupping experiment.

All examinations were performed in the Rehabilitation Engineering Laboratory at the University of Illinois at Urbana-Champaign. The participants finished four tests; each time last 20–30 min at four different days to prevent the carryover effort. All participants were required to acclimate themselves to the room temperature for 30 min before the experiment. Pre-cupping ultrasound images over the triceps belly of each participant were measured 3 times in the relaxed, supine position with the elbow in full extension, the forearm in full pronation, and the wrist in the neutral position (Figure 1). Then, one of four cupping therapy protocols was applied on the center of SJ12 acupoint. The cupping pressure and duration was pre-determined and assigned to the participant based on a counter-balanced design to minimize the order effect (Table 1). The allocation of a subject was based on the order a participant who signed up for this study. This allocation was based on the time that a participant indicated an interest for this study and was not dependent on any other factors. Then, the cup was removed and the ultrasound images were measured 3 times again (post-cupping).

### Muscle stiffness measurements

**FIGURE 2:**
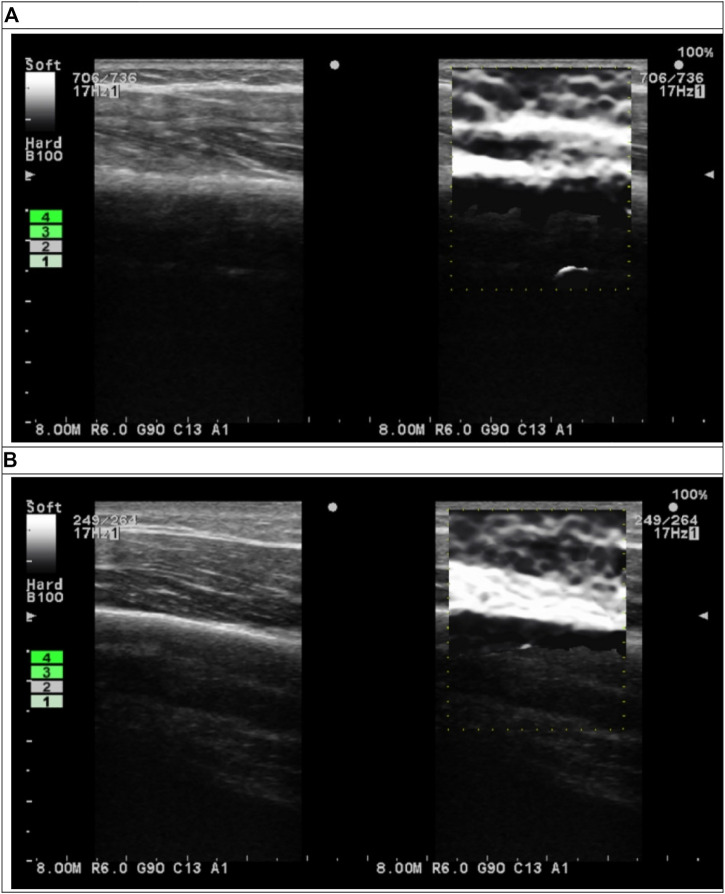
B-mode (left subfigures) and elastographic (right subfigures) ultrasound images of triceps **(A)** before and **(B)** after cupping therapy.

B-mode and elastography ultrasound images of the triceps were measured using an elastography ultrasound device (ProSound A7; Hitachi Healthcare Americas, Twinsburg, OH). Because the limb muscle may be thicker than 6 cm, 17-MHz ultrasound signal was selected for this study. The ultrasound probe has a frequency of 17–21 MHz (UST-5412; Hitachi Health care Americas). The device provides a strain graph and an indication of the tissue compression between consecutive ultrasound frames (Figure 2). A value of three or four indicates sufficient compression and appropriate speed of compression for the desired contrast in strain within the region of interest (ROI). This indicator was used to improve the consistence of the compression procedures for all measurements. In this study, the compression indicator was chosen at four for all strain elastographic measurements. All ultrasound images were saved as Digital Imaging and Communications in Medicine (DICOM) files.

### Data analysis

The DICOM files of B-mode ultrasound images were used to identify the location of humerus of each image. Then, the pixel location of humerus on the B-mode images was used to define ROI on elastographic images (Figure 2). In the elastogram, softer tissue is more compressible and has a higher strain ratio. Stiffness is inversely proportional to the measured strain when loading is kept the constant. Thus, strain ratio values were inversely converted to the stiffness values. The semi-quantitative elastogram images were converted to the gray level for the quantification purpose. Each pixel in the gray level images represented stiffness ranged from 0 to 255 (hardest). The bone tissue was used to validate the gray scale. The ROI in this study was defined as a rectangular area from the skin surface to the humerus in each image. The width of ROI was defined as the central 80% of the ultrasound image to avoid edge distortion. The depth was defined from the skin to the edge of humerus. The mean value of all pixels within the ROI were computed to represent the stiffness. The ROI was evenly divided into the superficial (skin, fat, subcutaneous and muscle) and deep (muscle) layers. The mean value of all pixels within the superficial and deep layers were computed for each participant. The value of stiffness is represented by dividing the post-cupping stiffness to pre-cupping stiffness to each condition of each participant (i.e. normalized stiffness). Matlab and the Image Processing Toolbox (2019R, MathWorks, Inc., Natick, MA) were used to perform calculations. Two-way repeated measures analysis of variance (ANOVA) was used to examine the main effects of the pressure and duration factors and the interaction effect between the pressure and duration factors. The test of sphericity was used to examine whether the assumption of a normal distribution was met. For the post-hoc comparisons, the paired-t test with the Bonferroni correction was used to compare the difference between two conditions if the main effect exists. The significance level was set at *p* < 0.05. SPSS (Version 29, IBM) was used to implement all statistical tests.

## Results

In this study, 12 healthy, young participants (5 male and 7 female) were recruited from the students and the staff of University of Illinois. The demographic data were as follows: age 25.42 ± 4.9 years, body height 1.7 ± 0.1 m, body weight 75.1 ± 18.7 kg, body mass index 25.2 ± 4.4 kg/m^2^, arm circumference 28.9 ± 3.6 cm, systolic blood pressure 112.8 ± 13.7 mmHg, diastolic blood pressure 69.3 ± 8.6 mmHg, heart rate 74.4 ± 7.8 beats/min, and eight Asians and Asian Americans and four Caucasians.

**FIGURE 3:**
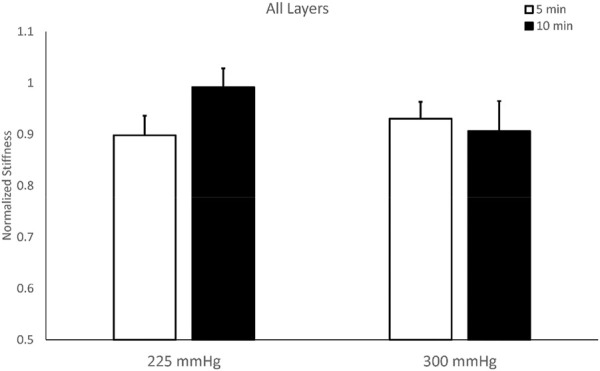
Comparisons of overall stiffness of the triceps muscle after cupping therapy at 225 and 300 mmHg for 5 and 10 min. Three of four cupping protocols have lower post-cupping stiffness compared to pre-cupping stiffness, except cupping therapy at 225 mmHg for 10 min.

The stiffness of the overall layer of the triceps shows a significant decrease in the three protocols (225 mmHg for 5 min, 300 mmHg for 5 min, and 300 mmHg for 10 min), but not in the protocol of 225 mmHg for 10 min in Figure 3. The two-way repeated measures ANOVA indicates that there are no interactions between the pressure and duration factors on normalized stiffness of the overall layer (F = 2.405, *p* = 0.149) (Table 2). The test of sphericity indicates no violation of the assumption. Normalized stiffness of triceps muscle is 0.898 ± 0.038 (225 mmHg for 5 min), 0.930 ± 0.033 (300 mmHg for 5 min), and 0.906 ± 0.058 (300 mmHg for 10 min) of pre-cupping stiffness, and is 0.992 ± 0.037 (225 mmHg for 10 min) of pre-cupping stiffness (Figure 3).

**TABLE 2:** Statistical results of the two-way ANOVA with repeated measures.

		F Values	*p* Values	Effect size
Pressure	Overall	1.057	0.326	0.088
	Superficial	1.838	0.202	0.143
	Deep	0.205	0.660	0.018
Duration	Overall	0.403	0.538	0.035
	Superficial	3.284	0.097	0.230
	Deep	7.650	0.018*	0.410
Pressure x Duration	Overall	2.405	0.149	0.179
	Superficial	0.243	0.632	0.022
	Deep	0.507	0.491	0.044

**FIGURE 4:**
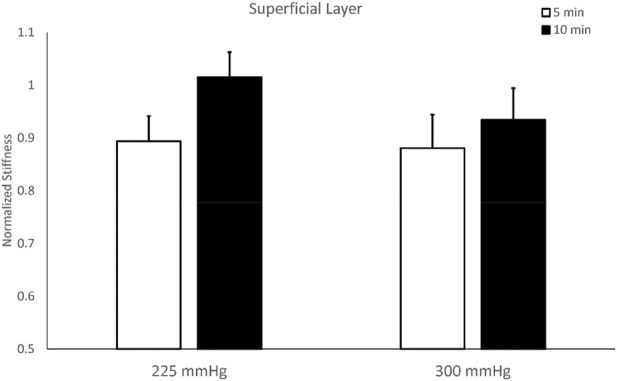
Comparisons of stiffness of the superficial layer of the triceps muscle after cupping therapy at 225 and 300 mmHg for 5 and 10 min. For the superficial layer, 5-min cupping appears more effective on reducing stiffness compared to 10-min cupping.

The stiffness of the superficial layer of the triceps did not show significant differences among four protocols. The two-way repeated measures ANOVA indicates there are no interactions between the pressure and duration factors on normalized stiffness of the superficial layer (F = 0.243, *p* = 0.632), and there is a trend on the duration effect (F = 3.284, *p* = 0.097, effect size = 0.230) (Table 2). The test of sphericity indicates no violation of the assumption. Under 225 mmHg, normalized stiffness of the triceps after 5-min cupping (0.893 ± 0.048) is lower than 10-min cupping (1.014 ± 0.048, *p* = 0.051). Under 300 mmHg, the stiffness of the triceps after 5-min cupping (0.880 ± 0.064) is lower than 10-min cupping (0.934 ± 0.060, *p* = 0.523) (Figure 4; Table 2).

**FIGURE 5:**
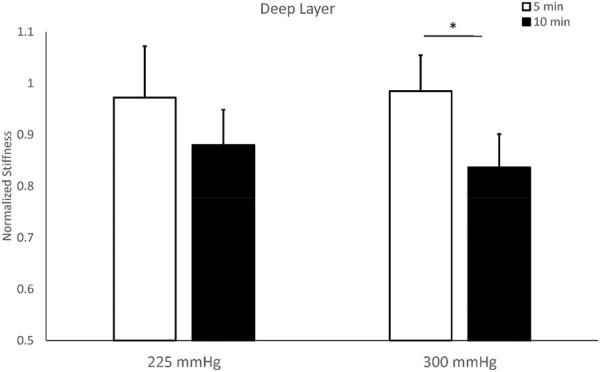
Comparisons of stiffness of the deep layer of the triceps muscle after cupping therapy at 225 and 300 mmHg for 5 and 10 min. For the deep layer of triceps, 10-min cupping therapy induces a significant decrease in stiffness compared to 5-min cupping. Compared to pre-cupping stiffness of each protocol, 10-min cupping effectively reduces post-cupping stiffness of the deep layer of triceps. * indicates *p* < 0.05.

The stiffness of the deep layer of the triceps shows a significant difference between 5-min (0.984 ± 0.070) and 10-min cupping (0.886 ± 0.064) under 300 mmHg (*p* < 0.05). The two-way repeated measures ANOVA indicates there are no interactions between the pressure and duration factors on normalized stiffness of the deep layer (F = 0.507, *p* = 0.491), and there is a significant main effect on the duration factor (F = 7.650, *p* = 0.018, effect size = 0.410) (Table 2). The test of sphericity indicates no violation of the assumption. Under 300 mmHg, normalized stiffness of the triceps is significantly higher under 5 min cupping (0.972 ± 0.10) compared to 10-min cupping (0.879 ± 0.069, *p* < 0.05) (Figure 5; Table 2).

## Discussion

This study provides the first evidence showing the efficacy of different intensities of cupping therapy on reducing muscle stiffness. Our results demonstrate that cupping therapy at -300 mmHg for 10 min is more effective on reducing stiffness (normalized stiffness in this study) of the deep layer of the triceps compared to 5-min cupping. These results confirm our hypotheses that responses of muscle stiffness to cupping therapy are affected by the magnitude and duration of negative pressure. The finding of this study supports the need for establishing the dose-response relationship of cupping therapy for improving clinical outcomes of cupping therapy.

When comparing the effect of cupping therapy on the superficial (i.e. the skin, subcutaneous tissue, fascia and fat) and deep layers (i.e. fascia and muscle) of the triceps, 10-min cupping at −300 mmHg was more effective on reducing normalized stiffness of the deep layer of the triceps. This appears consistent with the finding from one of our previous studies assessing effects of various cupping sizes on reducing muscle stiffness and on the deep layer of the muscle was more sensitive to cupping therapy for a significant decrease in muscle stiffness (Jan et al., 2021). It is generally agreed that the skin and fat tissue is relatively incompressible compared to the muscle. Thus, cupping therapy should be more effective on reducing the deep layer of the muscle, that is, mainly muscles rather than the incompressible skin and fat tissue of the superficial layer (Rodrigues and EEMCO, 2001; Jan et al., 2021). It has been confirmed that using shear-wave elastography to demonstrate a decreased muscle shear modulus when the skin on the muscle was removed (Yoshitake et al., 2016). The skin and fat tissue have a relatively uncompressible mechanical property and may not significantly respond to cupping therapy (Jan et al., 2013; Jan et al., 2021).

In addition to confirm that cupping therapy could reduce stiffness of the muscle using elastographic ultrasound in this study, we further demonstrate that the difference of response of normalized muscle stiffness during different intensities of cupping therapy. Cupping therapy at −300 mmHg for 10 min reduces the stiffness of the deep layer of the triceps compared to 5-min cupping at the same pressure. We further demonstrate that under the same duration of cupping therapy, a higher absolute value (−300 mmHg) of negative pressure is more effective on decreasing muscle stiffness compared to a lower absolute value (−225 mmHg) of negative pressure after cupping therapy (main effect of the duration factor, F = 7.650, *p* = 0.018, and effect size = 0.410). These results provide the evidence for the commonly used doses of cupping therapy on reducing muscle stiffness. This could also support why a less experienced Chinese medicine doctor could not deliver an effective cupping therapy because the use of fire cupping technique may not be easy to reach sufficient negative pressure inside the cup.

Regarding the duration, we demonstrated that a shorter duration of cupping therapy is more effective on the superficial layer and a longer duration of cupping therapy is more effective on the deep layer (main effect of the duration, F = 3.284, *p* = 0.097, and effect size = 0.230). This seems consistent with the finding from one of our previous studies on assessing skin blood flow (SBF) response to cupping therapy. Wang et al. demonstrated that under the same negative pressure, a shorter duration (5 min) may cause a larger peak and total SBF compared to a longer duration (10 min) after cupping therapy (Wang et al., 2020). In this study, we further confirmed that the shorter duration (5 min) was more effective on reducing stiffness of the superficial layer, including the skin. This finding has clinical implications because the use of cupping therapy with various durations can be used to target various depth of soft tissue underneath the cupping cup. Compared with muscle stiffness, the response of skin blood flow to cupping therapy is more sensitive, even a lower absolute value (−225 mmHg) of negative pressure after cupping therapy can increase skin blood flow. Adequate mechanical force, such as negative pressure caused by cupping therapy, is required for the force-deformation of deep tissue (muscle), compared with the change of microvasculature of surface tissue (skin). Because the muscle is a viscoelastic material, its mechanical response to cupping therapy is a time-dependent response. Thus, a longer time may be needed to induce a significant decrease in muscle stiffness. However, the definitive relationship between the duration of cupping therapy and the response from the soft tissue requires more research.

Using the non-invasive device, elastographic ultrasound, the muscle stiffness response to four different intensities of cupping therapy were quantified for the first time in the literature. Elastographic ultrasound has provided a quantifiable spatial representation of elasticity in the form of an elastogram by showing biomechanical changes in tissues following application of physical stress (Creze et al., 2018). Elastography has gained an important role in the diagnostics, staging and follow-up of numerous diseases and is now part of routine examination for soft tissues imaging, such as breast cancer, liver fibrosis thyroid, prostate, kidney and lymph node (Sigrist et al., 2017). Among its various indications, elastography is especially promising in evaluating the musculoskeletal system to assess efficacy of various interventions, including cupping therapy (Bilston and Tan, 2015; Paluch et al., 2016). Strain elastography, as the earliest elastography technology, measures tissue stiffness by applying external tissue pressure. It can be further divided into two groups by the method of tissue excitation (external manual excitation or excitation with internal physiological movement) (Ozturk et al., 2018). Manual compression works fairly well for superficial organs such as the breast and muscle, but is challenging for assessing elasticity in deeper located organs, such as the liver and kidney (Kim et al., 2016; Ozturk et al., 2018). One of the disadvantages of the strain elastography is that the generation of mechanical stress by hand, which means technique is obviously extremely operator dependent. Therefore, it represents only a semi-quantitative approach and is not able to quantify exact tissue stiffness. However, the strain ratio may serve as a substitutional parameter of stiffness (Schmalzl et al., 2017). In this research, strain elastography was chosen to evaluate the response of muscle to different pressures and durations of cupping therapy. Strain ratio data have been used to quantify the muscle stiffness that is highly relevant to sports performance and muscle fatigue and soreness. Research found that muscle stiffness is linearly related to both active and passive muscle forces (Hug et al., 2015). For athletes, force-deformation characteristics of the lower limb muscles have been associated with athletic performance and may modulate the risk of injury (Brazier et al., 2019).

Cupping therapy is a mechanical force based intervention just like stretching and massage by applying force to soft tissue. These interventions have been popular for reducing muscle stiffness and soreness in athletes (Hou et al., 2021b; Hou et al., 2021c). This study provides the first evidence showing the effect of pressures and durations of cupping therapy on muscle stiffness responses. Cupping therapy can decrease muscle stiffness under appropriate cupping intensities that may improve sports performance and reduce muscle fatigue and soreness. Based on our results, the duration parameter is particularly important on targeting the various depth of soft tissue; a shorter duration (e.g. 5 min) may be effective on the superficial layer (*p* = 0.097) and a longer duration (e.g. 10 min) is effective on the deep layer of muscle (*p* = 0.018). Based on our findings, clinician should be aware that patients who are overweight or obese may need to use a relatively longer duration of cupping therapy to reach to the deep layer. However, a person who is skinny should be applied a shorter duration of cupping therapy to induce more skin blood flow at the superficial layer. However, these clinical scenarios should be examined for evidence. Based on our study, inappropriate intensity of cupping therapy may not benefit the musculoskeletal system, which may partly explains the conflicting results of cupping therapy on managing musculoskeletal disorders.

There are limitations of this study. First, we only focus on the effect of cupping therapy on the triceps muscle. Our results may not be generalized to different muscles (e.g., medial gastrocnemius muscle) with different characteristics (e.g., different thickness and stiffness levels). Future studies may use our protocols to examine biomechanical responses of lower extremity muscles to various intensities of cupping therapy to develop specific dose-response relationship for the muscle. Second, this study was conducted in a homogenous group of participants with similar body mass index. The results may not be generalized to people who are overweight or underweight because of the potential influence of fat tissue in response to cupping therapy. Also, we recruited both genders in this study. It is unclear whether the gender effect would affect the efficacy of cupping therapy on reducing muscle stiffness. Third, the authors measured muscle stiffness responses using strain elastography. Future studies may combine strain elastography and shear wave elastography to measure muscle stiffness and shear modulus in response to cupping therapy. Fourth, strain elastography requires the operator to apply loading to induce deformation of soft tissue. Such procedures may involve the variations of the magnitude of loading for calculating tissue strain. This step may become a potential source of variability in measured tissue stiffness. Last, we assessed the efficacy of cupping therapy on reducing muscle stiffness in healthy people. It is unclear whether our results could be reproduced in patients with muscle soreness.

## Conclusion

In this study, B-mode and elastographic ultrasound was used to assess stiffness of the triceps after various negative pressures and durations of cupping therapy for the first time in the literature. Our findings demonstrate that 10-min cupping at −300 mmHg is more effective on reducing stiffness of the deep layer compared to 5-min cupping. Our study provides the first evidence showing various negative pressures and durations of cupping therapy could affect muscle responses. The finding of this study indicates a need of establishing the dose-response relationship of cupping therapy for improving clinical outcomes.

## Data Availability

The original contributions presented in the study are included in the article/supplementary material, further inquiries can be directed to the corresponding authors.
